# Supplementary material for: Eurotium cristatum Ameliorates Glucolipid Metabolic Dysfunction of Obese Mice in Association with Regulating Intestinal Gluconeogenesis and Microbiome
Source: Foods. 2025 Dec 12;14(24):4273. doi: 10.3390/foods14244273 (PMC12733180; doi:10.3390/foods14244273)
Supplement: Supplementary file 1 [file foods-14-04273-s001.zip › foods-3999852-supplementary.pdf]

***Eurotium cristatum* ameliorates glucolipid metabolic dysfunction of obese mice in association with regulating intestinal gluconeogenesis and microbiome**

Weirong Yang[a], Ning Han[b], and Xiangnan Zhang[b,]*

[a]*Faculty of Science, The University of Technology Sydney, Sydney, 2007, Australia*

[b]*College of Food Engineering and Nutritional Science, Shaanxi Normal University, Xi'an 710119, China*

Corresponding author

E-mail address: zhangxiangnan@snnu.edu.cn (X.N. Zhang)

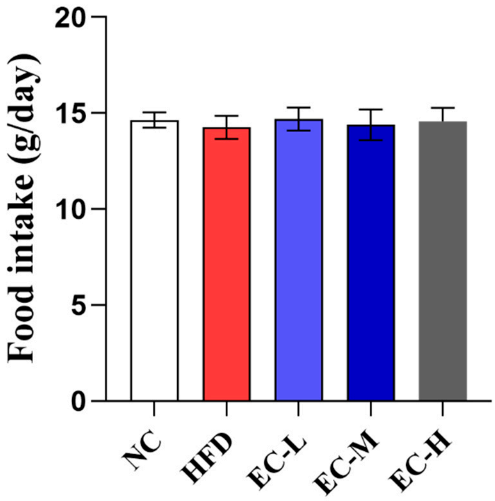

Figure S1. Food intake. (*) $p < 0.05$ and (**), $p < 0.01$ vs. HFD group.
